# Spatial Dynamics of Harbour Porpoise *Phocoena phocoena* Relative to Local Hydrodynamics and Environmental Conditions

**DOI:** 10.1002/ece3.71334

**Published:** 2025-05-07

**Authors:** Robert Mzungu Runya, Chris McGonigle, Rory Quinn, Morgane Pommier, Christian Armstrong

**Affiliations:** ^1^ School of Geography and Environmental Sciences Ulster University Co. Derry UK; ^2^ Marine and Freshwater Research Centre Atlantic Technological University Galway Ireland; ^3^ Scottish Association for Marine Science Oban, Scotland UK

**Keywords:** conservation, habitat use, harbour porpoises, hydrodynamic, remote sensing, species distribution models

## Abstract

Understanding the spatial dynamics of harbour porpoise ( *Phocoena phocoena* ) is crucial for effective conservation and management. The study presents a multidisciplinary approach to modelling and analysing the site occurrence and habitat use of *Phocoena phocoena* within the Skerries and Causeway Special Area of Conservation (SAC), identifying areas where they were seen surfacing and/or spending the most time. Using data derived from multibeam echosounders (MBES), particle size analysis of sediments, hydrodynamic modelling, and theodolite tracking observations, the study examines the influence of local hydrodynamics and environmental conditions on the spatial distribution of harbour porpoises. Kernel density analysis of 451 porpoise sightings over an 11‐day survey demonstrated that dense clusters and higher aggregations occurred within ~500 m of the shoreline. Generalised Additive Models (GAMs) identified slope, aspect, backscatter intensity and sediment grain size as the most significant environmental predictors, accounting for 47.6% of the deviance in harbour porpoise distribution. Porpoises' occurrence was particularly spatially coincident with coarser sediments (4.25–5 mm), and their distribution was highly concentrated around headlands, shoreline and within a 3‐h window before and after high water. Overall, these findings highlight the dynamic nature of harbour porpoises' use of habitat in space and time, with models predicting a high probability of porpoise encounters (> 0.6) nearshore, particularly in headland areas characterised by local flow acceleration and coarser seabeds. The study presents a robust workflow for developing a porpoise‐specific monitoring program. By leveraging multidisciplinary methodological approaches, the study provides a scientific basis for refining marine conservation measures, delivering long‐term protection for harbour porpoise habitats under existing legal and management frameworks both within and beyond the SAC boundaries.

## Graphical abstract

This study uses a multidisciplinary approach to model the habitat use and site occurrence of harbour porpoises, identifying areas where they were seen spending most time in the Skerries and Causeway Special Area of Conservation. The results show that porpoises are most concentrated within 500 m of the shoreline, particularly near headlands with coarse sediments and during specific tidal phases. The study highlights key environmental factors influencing porpoise distribution and emphasizes the need for targeted and strategic management of critical areas to support the protection of harbour porpoises and wider biodiversity.

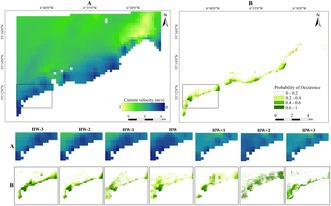

## 1 Introduction

The distribution of a species in the marine environment is well known to be a function of its interaction with prevailing biophysical factors (Calvert et al. 2018; Benjamins et al. 2017, 2016; Hays et al. 2016). However, anthropogenic activities and climate change threaten the diversity and distribution of marine species and habitats (Jefferson et al. 2021; Halpern et al. 2019). While quantifying human impacts is crucial for conservation efforts, understanding the environmental factors that influence the range and distribution of a species requires targeted ecological assessments (Runya et al. 2024; Jones et al. 2014; Booth et al. 2013). Multibeam echosounder (MBES) data sets are often utilised for broad‐scale marine surveys (McGeady et al. 2023; Menandro et al. 2022; Runya et al. 2021; Brown et al. 2019). Despite recent advancements, acquisition and analysis of MBES data are optimised for geological (Giglio et al. 2022), hydrographic and archaeological use with limited ecological applications. The lack of comparable spatial and temporal resolutions between remote sensing and ground truth data, and backscatter calibration hinders the use of these MBES data sets (Runya et al. 2024, 2021; Brown et al. 2019; Lecours et al. 2016). In addition, due to the prohibitive cost of marine surveys, marine scientists and hydrographers have developed a practice of collecting and analysing data sets of multidisciplinary nature and the use of historical data. This in turn enables scientists to optimise acquisition platforms in terms of cost, time and aid opportunistic analysis and repurposing of archival data to provide credible scientific evidence needed for marine monitoring and management (Runya et al. 2024). However, these data require that they be collected at appropriate scales that fully account for the environmental variability and effectively address management needs (Benjamins et al. 2017; Tett et al. 2013).

Recently, there has been an increased scientific effort in the use of multidisciplinary analytical approaches for monitoring marine mammals such as harbour porpoises due to their conservation status and charismatic nature (Benjamins et al. 2017). Current literature on harbour porpoises ( *Phocoena phocoena* ) has predominantly focused on their habitat usage, range and distribution (Benjamins et al. 2017; Jones et al. 2014; Booth et al. 2013). The harbour porpoise, *P. phocoena* , is smaller in size than other cetaceans and prevalent throughout temperate Europe, including the North Atlantic part of Ireland and the United Kingdom. The population of this species has been subjected to various anthropogenic threats and pressures related to bycatch or strandings from gillnets (Irvine et al. 2024), chemical pollution (Williams et al. 2021, 2020), noise pollution from heavy maritime traffic (Findlay et al. 2024), offshore infrastructure, oil and gas extraction, marine renewable energy generation, coastal development and increased sea level rise (Viquerat et al. 2014). The protection of this species is prescribed under the EC Habitats Directive (Annexes II and IV), CITES (Appendix II), Bern Convention (Appendix II) and Bonn Convention (Appendix II). Also, the Agreement on the Conservation of Small Cetaceans of the Baltic, North East Atlantic, Irish and North Seas (ASCOBANS) of 1992 by European countries, including the United Kingdom, provides for its protection. This agreement supports the protection of specific areas, monitoring, research, pollution control and public awareness creation regarding dolphins and harbour porpoises. Furthermore, the Wildlife and Countryside Act 1981 and the Wildlife Order 1985 of Northern Ireland (United Kingdom) provide for the protection of all cetaceans including harbour porpoises. However, it is unclear what specific hydrodynamic and environmental variables are linked to their geographical distribution, occurrence and use of space in the Irish Sea and North Atlantic Ocean (Bouveroux et al. 2020; Sparling et al. 2015).

For harbour porpoises, their spatial and temporal distribution is strongly influenced by prey availability, which in turn is linked to various static and dynamic environmental variables (van Beest et al. 2018). While direct measurements of prey distribution remain challenging, these environmental variables (e.g., tidal features, seabed composition, bathymetric features) serve as useful proxies for identifying areas of high foraging potential (Schaffeld et al. 2016). The abiotic proxies influence the aggregation of various prey species, such as sandeels, Atlantic herring, whiting, cod, squids and shrimps known to be the key part of the harbour porpoise diet (Stedt et al. 2024; Ransijn et al. 2021; Greene et al. 2020). Consequently, models rely on these environmental variables to predict porpoise distribution while acknowledging the complex and dynamic interactions between prey materials and environmental conditions. Harbour porpoises are known to be opportunistic predators and are able to switch their behaviour to specific foraging conditions, giving them access to a wide range of prey resources (Stedt et al. 2024; Elliser et al. 2020). These porpoise–prey–environment interactions are mostly non‐linear (Benjamins et al. 2017; Jones et al. 2014; Marubini et al. 2009). Specifically, the spatiotemporal patterns in porpoise occurrence and habitat usage have been linked to various factors such as tidal stream environment, especially those adjacent to land features like headlands and narrow straits (Lewis et al. 2015; Pierpoint 2008), characterised by high flow speeds (Benjamins et al. 2016; Davies et al. 2012). Headlands support the formation of upwelling features, fronts and eddies, resulting in higher prey aggregation, which further enhances foraging opportunities compared to other places (Greene et al. 2020; Benjamins et al. 2015; Heinänen and Skov 2015). In terms of habitat usage, harbour porpoises spend 75% of their time hunting alone or in groups, exploiting tidal energetic regions that allow prey aggregation and greater foraging options (Schaffeld et al. 2016). Enhanced tidal activity circulates prey (e.g., fish, squids, shrimps), offering excellent foraging opportunities within these tidal environments (Jones et al. 2014) and prey sources (Rojano‐Doñate et al. 2024; Ransijn et al. 2021), which can indirectly be used to infer porpoise distribution. Conversely, high porpoise densities have also been found in low‐flow conditions, which could be due to topographic influence rather than enhanced foraging options (Booth et al. 2013).

Similarly, interaction between seabed roughness and underwater currents is known to generate complex vertical currents that potentially enhance porpoise foraging efficiency (Waggitt et al. 2018). In shallow depths, seabed roughness may impact marine animal presence (Lieber et al. 2018). Seabed roughness is a direct derivative of substrate composition, which is an indicator of the physical and biological processes in the marine environment (Montereale Gavazzi et al. 2016). Typically, heterogeneous substrates support the occurrence of higher prey densities (Benjamins et al. 2017). Meanwhile, local terrain and hydrodynamic conditions may influence the current speed at a site, aiding animal movement at a low energy cost (Heinänen and Skov 2015; Milne et al. 2013). For instance, hydrodynamic forcing acting over shallow terrain can concentrate zooplankton and zooplanktivorous fish (e.g., sandeels) at the surface, making foraging materials more available for harbour porpoises in tidally active regions (Embling et al. 2013). Other environmental characteristics that influence harbour porpoise distribution include moderate depths (Heinänen and Skov 2015; Jones et al. 2014; Marubini et al. 2009), time relative to high water (Jones et al. 2014), slope and distance to shore (Booth et al. 2013), tidal regime (Nuuttila et al. 2017; Isojunno et al. 2012) among others. Distance to land could be indicative of the influence of tidal regimes, hydrodynamic features (e.g., upwelling and frontal systems) and prey sources on harbour porpoise distribution (Booth et al. 2013). Additionally, proximity to land may reflect sheltering effects that influence prey distributions and prey–predator interactions in dynamic coastal environments (Booth et al. 2013; Embling et al. 2010). Despite considerable mention in the literature, it is unclear which specific environmental variables are the most significant, with site‐specific studies providing variable accounts of porpoise–environment relationships.

This study presents a multidisciplinary analysis, integrating modelling of extant data repurposed for the monitoring of harbour porpoise *P. phocoena* within the Skerries and Causeway Special Area of Conservation (SAC). We use local hydrodynamic (predicted depth‐averaged current velocity, tidal height) and environmental variables (MBES bathymetry and harmonised backscatter, bathymetry derivatives, predicted sediment grain size) as abiotic proxies to model the site occurrence and use of space of harbour porpoises, identifying areas where they were seen surfacing and/or spending the most time. The study objectives are: (i) to produce a substratum map of Skerries and Causeway SAC using new data and a modelling approach, (ii) to determine the explanatory power of environmental variables influencing the relative density of harbour porpoises' sightings, (iii) to identify the most important variables influencing harbour porpoises' density, and lastly (iv) to model the spatial distribution and probability of occurrence of harbour porpoises with respect to tidal phase.

## 2 Methodology

### 2.1 Study Area

**FIGURE 1:**
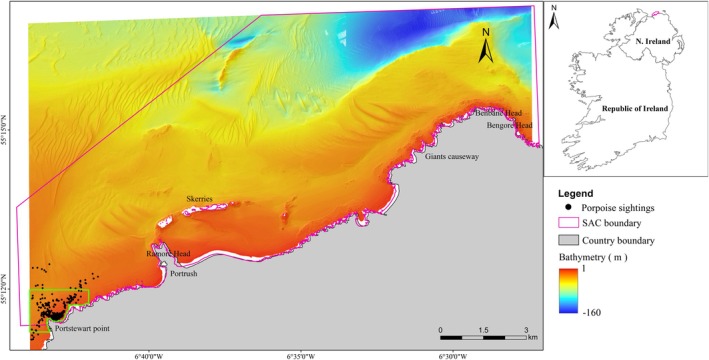
A map of the study area indicating the bathymetry gradient and harbour porpoise sighting positions within the boundary of the Skerries and Causeway SAC. The outline in green is the region of interest for Figure 4.

The Skerries and Causeway SAC covers 43.96 km^2^ off the north coast of Northern Ireland, in the South Malin Sea (Figure 1). The SAC was originally proposed as a Site of Community Importance (SCI) in 2012 and treated as designated (with legal protection measures in place) after formal acceptance by the European Union in 2013 (JNCC 2025). It was officially selected for designation as an SAC in 2017 under the EC Habitats Directive (SAC EU Code: UK0030383) based primarily on the presence of Annex I habitats: reefs (1370), sandbanks (1110) that are mostly submerged by seawater and submerged or partially submerged sea caves (8330) and Annex II species, harbour porpoises ( *P. phocoena* ) using the site for foraging and movement (JNCC 2022). The region is also rich in other non‐qualifying marine life, including the seagrass *Zostera marina* , and seabirds indicated by the presence of nesting grounds among other faunal communities (DAERA 2022; Erwin et al. 1990). Within the SAC, the Skerries Islands, formed from a Dolerite sill, stretch east of Portrush town off the coast of County Antrim (DAERA 2022; INFOMAR 2008). The SAC is bounded to the west by Ramore Head, to the east by Bengore Head and to the north by a reef often known as the ‘Ridges’ (Plets et al. 2012).

As part of the SAC designation process, a substrate benthic map of the area was produced to help define the SAC boundaries and identify the various substrate representations (Plets et al. 2012). This substrate map was produced using high‐resolution MBES bathymetry, bathymetry derivatives (e.g., slope, Bathymetric Position Indices), uncalibrated backscatter as well as historical ground truthing data comprising particle size and sediment type data (Plets et al. 2012). This mapping exercise identified the seabed within the SAC as being comprised of bedrock, rocky reefs, scoured bedrock with a veneer of mobile debris, coarse sediments, mixed sediments and sand (fine, medium and coarse).

The marine habitats inside the SAC are constantly subjected to the warm Gulf Stream and strong currents from the North Channel as well as persistent exposure to North Atlantic Ocean waves and surge (Plets et al. 2012). In certain areas, the SAC receives freshwater input from the Bann and Bush rivers. Furthermore, in the Skerries region, the southern Malin shelf waters and the North Channel of the Irish Sea are separated from each other by the Islay front (to the North‐west) and a salinity front to the east. This process provides higher sea temperatures than the Irish coast east of Bengore Head (Gowen et al. 1998). Meanwhile, the development of eddies in the bays and rip currents under surrounding rocks at the end of the bays is visible close to the beach (Carter 1991).

### 2.2 Data Acquisition and Processing

#### 2.2.1 Multibeam Data

In this study, legacy MBES data were utilised. These data were collected as part of the Joint Irish Bathymetry Survey (JIBS) by the Marine Institute and the Maritime and Coastguard Agency (Plets et al. 2012). The echosounder data were acquired with dual‐head Kongsberg EM3002 and EM710 and Reson 7125 MBES systems, deployed from RV *Jetstream*, RV *Victor Hensen and* RV *Meridian*.

##### 2.2.1.1 Bathymetry

Ungridded ASCII bathymetry data were processed, gridded and exported as an ArcGIS ASCII file in QPS Fledermaus (v.7.8.4) for this investigation. From the bathymetry grid, secondary derivatives were generated within ArcGIS (v.10.7.1). Using the Spatial Analyst extension, the slope angle and aspect were calculated in degrees to reflect the topographic steepness and compass orientation relative to the downward slope, respectively. In addition, the Benthic Terrain Modeler (BTM v.3.0) extension for ArcGIS was used to compute standardised fine‐and‐broad scale Bathymetric Position Indices (BPI) and Terrain Ruggedness Measure (VRM). The BPI is a secondary feature adopted from the Topographic Position Index (TPI), which is often employed in the terrestrial environment, and both are used to classify terrain features by comparing the elevation of a given point to its surrounding area. The TPI helps to identify various land features such as valleys, flat plains and ridges, whereas in the marine domain, the broad‐scale and fine‐scale BPI features help distinguish between larger and smaller seabed structures (e.g., reefs, sand waves, channels), respectively (Calvert et al. 2015; Plets et al. 2012), whose influence on hydrodynamics, sediment characteristics and prey aggregation can be linked to the distribution of biological communities (Lieber et al. 2018; Benjamins et al. 2017). The broad‐scale BPI was estimated with an inner radius of 10 cells and an outside radius of 100 cells, whereas the fine‐scale BPI was computed with an inner radius of 1 cell and an outer radius of 20 cells (Runya et al. 2024; Plets et al. 2012). In the meantime, VRM, which is a single measure of variation in slope and aspect, was computed using a neighbourhood size of 5 × 5 pixels to provide roughness in more localised locations. These bathymetric derivatives form a crucial element of terrain classification during benthic habitat mapping and seabed characterisation studies (Lecours et al. 2016; Calvert et al. 2015; Plets et al. 2012). Each raster mosaic was exported with a cell size of 1 × 1 m and projected to UTM zone 29 N.

##### 2.2.1.2 Backscatter

The raw data (*.all) were radiometrically and geometrically corrected in QPS Fledermaus Geocoder Toolbox (FMGT v.7.8.9), yielding three single backscatter mosaics that were exported as ArcGIS ASCII files for subsequent processing and analysis (Plets et al. 2012). These backscatter mosaics are frequently depicted as greyscale images indicating the relative strength of the backscatter signal, which offers information about the seafloor's roughness (Runya et al. 2024; Misiuk et al. 2020). The initial substratum map of Skerries and Causeway SAC prior to designation was created using uncalibrated backscatter mosaics (Plets et al. 2012).

The absence of calibration between repeat and overlapping surveys and between various multibeam data has historically restricted the application of backscatter data for seabed mapping and ecological applications (Misiuk et al. 2021, 2020). To correct for this, we applied a harmonisation technique that uses areas of mutual overlap between surveys to conduct a relative statistical calibration, also referred to as bulk shift (Misiuk et al. 2021, 2020; Hughes Clarke et al. 2008). The EM3002 data were selected as the reference (target) layer due to its broad spatial coverage and strong signal‐to‐noise ratio, and the EM710 and Reson 7125 data were the shift layers adjusted and afterwards harmonised using the linear model bulk shift function in R software.

#### 2.2.2 Sediment Grain Size Modelling

To aid in the classification of substrates, sediment grain size modelling was performed. First, known areas covered by bedrock were manually classified with a view to generate an analysis mask for the sedimentary component of the seafloor. In this investigation, sediment grain size modelling using a similar framework to that of Runya et al. (2021), with mean grain size from 25 grab samples, was used as the response variable. These grab samples were collected during a 2013 research cruise and analysed for particle size (Evans 2018). Three predictor variables, including harmonised backscatter, bathymetry and slope, were qualified to predict the mean sediment grain size using a Generalised Linear Model (GLM) of the Gaussian family with an identity link. To improve the robustness of the models, a leave‐one‐out cross‐validation (LOOCV) was employed in both model development and evaluation. The predicted surface of sediment grain size was imported into ArcGIS, and values were converted to substrate classes using the Folk sediment classification scheme (Long 2006). In addition, for each porpoise sighting record, the average sediment grain size within a 3 × 3 m window was calculated and exported for inclusion as an additional covariate in the subsequent analysis.

#### 2.2.3 Tidal Flow Data

Tidal data were downloaded from the British Oceanographic Data Centre (BODC) for the station gauge in Portrush. These tidal height records were used for our study site and were retrieved from this gauge between 6 August and 17 August, 2020. The tidal height data were then converted from 15‐min data to hourly values and all the values are relative to Admiralty Chart Datum (ACD). This data set yielded two predictor variables: the hours relative to Time to High Water (TtHW) and the tidal height (m) measurement.

#### 2.2.4 Hydrodynamic Modelling

**FIGURE A1:**
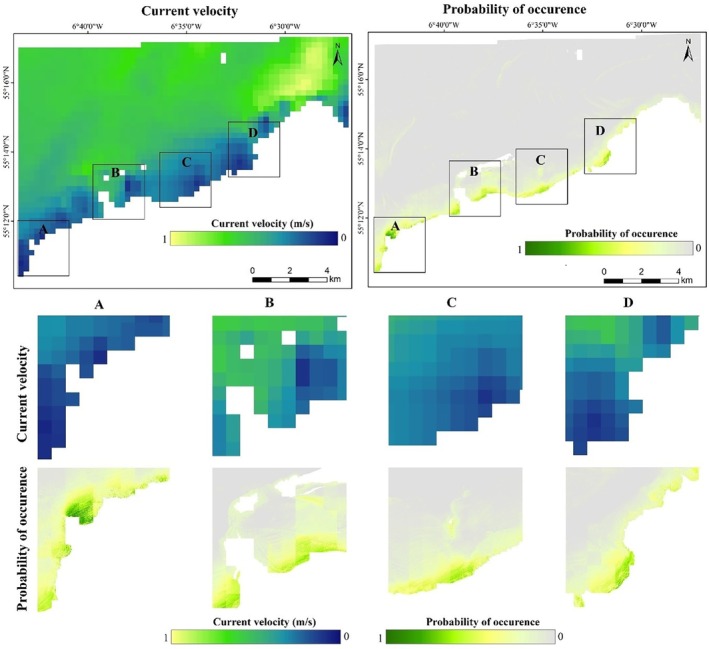
Average conditions for current velocity and probability of occurrence in four areas (A–D) showing the spatial dynamics in harbour porpoises' occurrence and site use.

**FIGURE B1:**
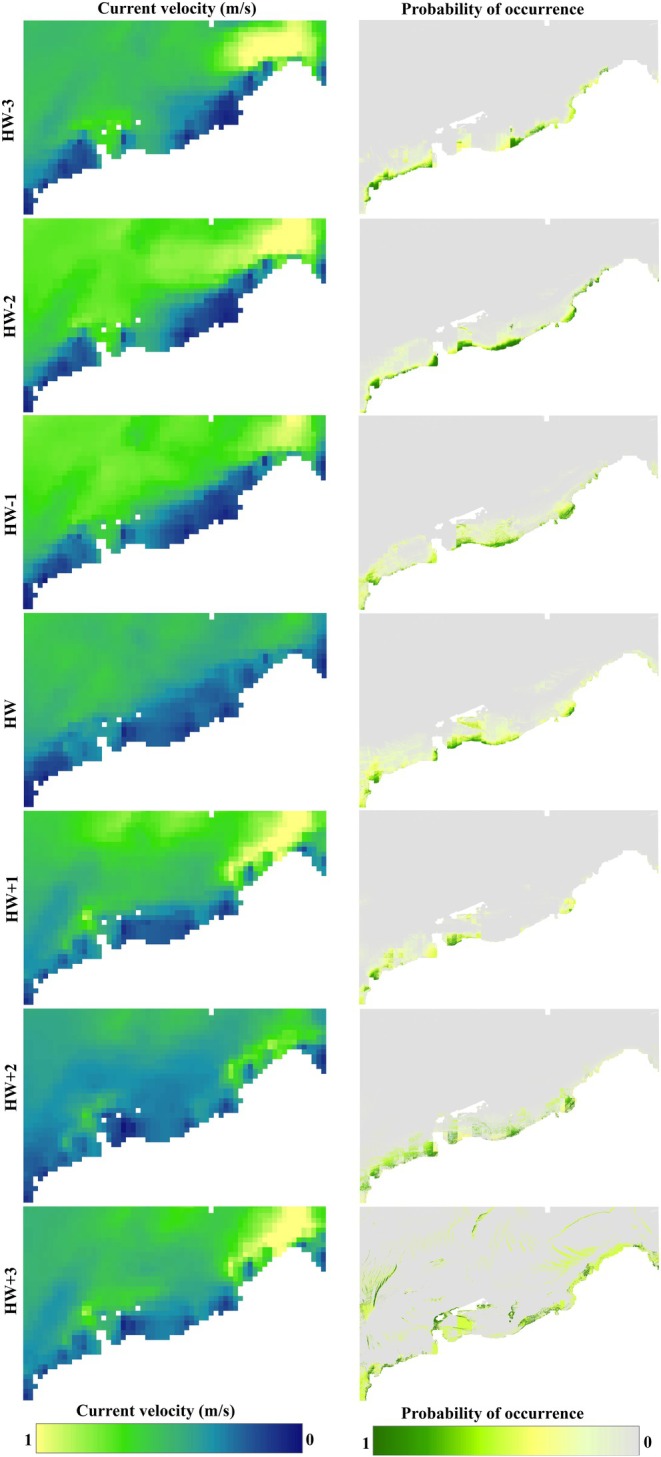
Hourly average conditions (for neap and spring tidal phases) between current velocity and modelled probability of occurrence of harbour porpoises in Skerries and Causeway SAC.

Hydrodynamic conditions were determined using a Delft‐3D model of depth‐averaged current velocity produced by the Scottish Association of Marine Sciences (SAMS). DELFT3D‐FLOW (tag: 59659) was used to broadly simulate the tidal flows surrounding the study site. A 239 × 131 depth‐averaged curvilinear grid was produced using the Arakawa‐C structure, adopting the Boussinesq assumption and assuming hydrostatic pressure. The model was nested within the TPXO Global Inverse Tidal Model (v.8.0) possessing a spatial resolution of 285.9 m. Uniform bottom friction was prescribed with a Chezy roughness of 65, and the free slip condition was implemented at closed boundaries. The model was run from 01 August 2020 (00:00:00 GMT) to 30 August 2020 (00:00:00 GMT). A model time step of 6 s ensured that the Courant–Friedrichs–Lewy condition, indicated by a Courant number < 1, was satisfied. Fourteen model outputs were produced: seven for spring and seven for neap tidal phases, with hourly time stamps ranging from −3 to +3 h relative to high water. These model outputs were exported as rasters (.tif) formats and resampled to 2 m. The models of neap and spring tidal phases were averaged to seven final models representing −3 to +3 hourly models relative to high water (Figure A1: Appendix A and Figure B1: Appendix B).

Water‐level data were used for model validation with sampling undertaken at Portrush (UK). Harmonic analysis was performed using t‐tide (Pawlowicz et al. 2002) to reproduce a tide‐only signal devoid of atmospheric pressure effects. The resultant signal was compared to the corresponding water‐level data from the model.

#### 2.2.5 Theodolite Tracking

The position, movement and behaviour of harbour porpoises were visually tracked over a period of 11 days when visibility conditions were good [low sea state (1–3 for porpoises), no fog, no rain] in the Skerries area using theodolite tracking stationed at 55.1890°–6.7203°. This data set was acquired by the Galway Marine Institute of Technology (GMIT) with the support of the Agri‐Food Biosciences Institute (AFBI) and Ulster University (UU) as part of a collaboration between the SeaMonitor and COMPASS projects and made available for the purpose of this study. During the tracking, a theodolite was utilised to measure the distance between harbour porpoises and acoustic receivers (55.1975°–6.7168°) with a horizontal reference at 55.1894°–6.7203°. The position of the animals was captured as often as possible while they were at the surface, with priority given to the individuals closest to the acoustic receiver, which in this case was used as a fixed reference point for theodolite tracking. Computing distance this way enables an accurate estimation of the detection range. Each pair of vertical and horizontal angles was measured, and time noted. It is important to note that this data set was initially collected to estimate detection range, with a primary focus on the acoustic receiver. In this present study, the data set is repurposed for a different objective, which may explain the methodological limitations highlighted in this paper. Ideally, the position of multiple groups of animals would have been recorded, rather than being limited to individuals closest to the acoustic receiver, allowing for a more detailed analysis.

Theodolite's external sight was used to determine their geographic positions, providing the observer a better view of the surrounding environment. Although this method facilitates tracking, it inherently affects the precision of angle measurement (Gailey et al. 2007). To account for this inherent variation in theodolite recording (referred here as a fix) accuracy, each point was assigned a precision rating between 1 and 4, where 1 denotes a precise fix (porpoise point through the telescope), 2 was a normal fix with a marker in the external sight used to point at the porpoise location amd 3 corresponded to an approximate fix (where the position was recorded using the external sight, but a few seconds after surfacing). Last, for measurements obtained from memory after the dive hence prone to error, a rating of 4 was given. However, grade 4 fixes were excluded from the analysis. These theodolite fixes or surfacing events are thereafter in this manuscript referred to as sightings.

### 2.3 Data Analysis

#### 2.3.1 Spatial Density Estimation

The spatial density of porpoise sightings within the study area was estimated using the Kernel Density function in ArcGIS' Spatial Analyst toolbox. This function computes distribution density per unit area (km^2^) from the point sighting records (*N* = 451) and generates a smooth heat map showing cluster variability comparable to high occurrence areas.

#### 2.3.2 Harbour Porpoise Data Analysis and Statistical Modelling

Standard data exploration procedures were applied to the data before any analysis was performed. These procedures included quality checks for the missing data, collinearity between covariates and data distribution in the form of boxplots. Additionally, collinearity between covariates and the relationship between the response variable and covariates were examined (Benjamins et al. 2017; Jones et al. 2014). The Pearson correlation coefficient was used to determine the strength of the link between the response variable (density of porpoise sightings relative to time) and the covariates. The study also used the Pearson correlation coefficient and the variance inflation factor (VIF) to check for collinearity between covariates. This was done in accordance with the recommendations given by Zuur et al. (2010) and applied in Runya et al. (2024) and Benjamins et al. (2017). As a minimum threshold for excluding collinearity, a correlation coefficient of 0.7 or higher between paired covariates and a VIF > 5 were used. In this instance, harmonised backscatter, bathymetry, slope, VRM, aspect, tidal height, TtHW, closest distance to the shoreline and predicted sediment grain size were qualified. It has been shown that if collinearity is not properly accounted for, this might result in inaccurate estimation of model coefficients as well as model accuracy (Zuur et al. 2010). The amount of time spent conducting the survey (or survey effort) and detecting porpoises was initially analysed using summary statistics.

Porpoise sighting density (response variable) was modelled using a Generalised Additive Model (GAM) of the Poisson family with a log link with respect to environmental variables. The porpoise sighting densities derived from theodolite records, were normalised for time‐varying water levels (time relative to high water) and computed by grouping observations into 10‐min bins to reflect their use of space (Jones et al. 2014; Simon et al. 2010). These GAM models were used to examine the dynamics of occurrence within Skerries and Causeway SAC with respect to local hydrodynamics and environmental variables. Average values within a 3‐by‐3 analysis window for each covariate relative to the position of harbour porpoises' sightings were determined. This study window was deemed appropriate to account for the environmental variability in relation to the spatial footprint and limited movement of harbour porpoises during foraging. The GAM analysis was performed using the ‘mgcv’ library (v.1.8‐31) in R. Smoothers of covariates were specified with thin plate regression splines (Jones et al. 2014). For all model covariates, the k (limiting the maximum degrees of freedom for each smooth) was restricted to *k* = 4, hence preventing model overfitting and excessive flexibility (Benjamins et al. 2017; Jones et al. 2014). The models' performance between univariate and multivariate models were undertaken using the common root mean square error (RMSE), adjusted *R* ^2^ value, *p* value and Akaike Information Criterion as summarised in the later section.

Predictive GAM models depicting the spatial distribution (use of space) and probability of occurrence of porpoises were generated using a series of libraries including sdm (1.1–8), raster (v.3.4‐10) and mgcv (v.1.8‐31) in R software. Data preparation involved generating pseudo‐absences in the ratio 20:1 (pseudo‐absences: presence) that allows for prediction based on distributions of presence and absence of a species. Pseudo‐absence records were generated using random sampling from the background (Barbet‐Massin et al. 2012; Wisz and Guisan 2009) and within the same spatial extent. This ratio was determined through a series of exploratory analyses that took processing time and output variability into account. Less than 5% variance in areal extent of the output between random replicates was observed. The model inputs comprised harmonised backscatter, bathymetry, slope, aspect and predicted depth‐averaged current velocity (continuous variables). Model formulation and validation were achieved using a cross‐validation method. The ability of the models to accurately predict the probability of occurrence was evaluated using the area under the curve (AUC), true skill statistic (TSS) and deviance values (Hazen et al. 2021; Barbet‐Massin et al. 2012; Engler et al. 2004). A prediction with AUC values of > 0.7 is considered good. The TSS accounts for the sum of models' sensitivity and specificity minus one (Barbet‐Massin et al. 2012). Because initial analysis showed that porpoise density was higher between 3 h before and after high water time, the 10‐min bins' observations for this period were selected. As a result, 14 predictive models showing the probability of occurrence and spatial distribution of harbour porpoises with respect to tidal phase (spring and neap tidal phases) between 3 h before and after high water were produced.

## 3 Results

### 3.1 Substratum Classification

**FIGURE 2:**
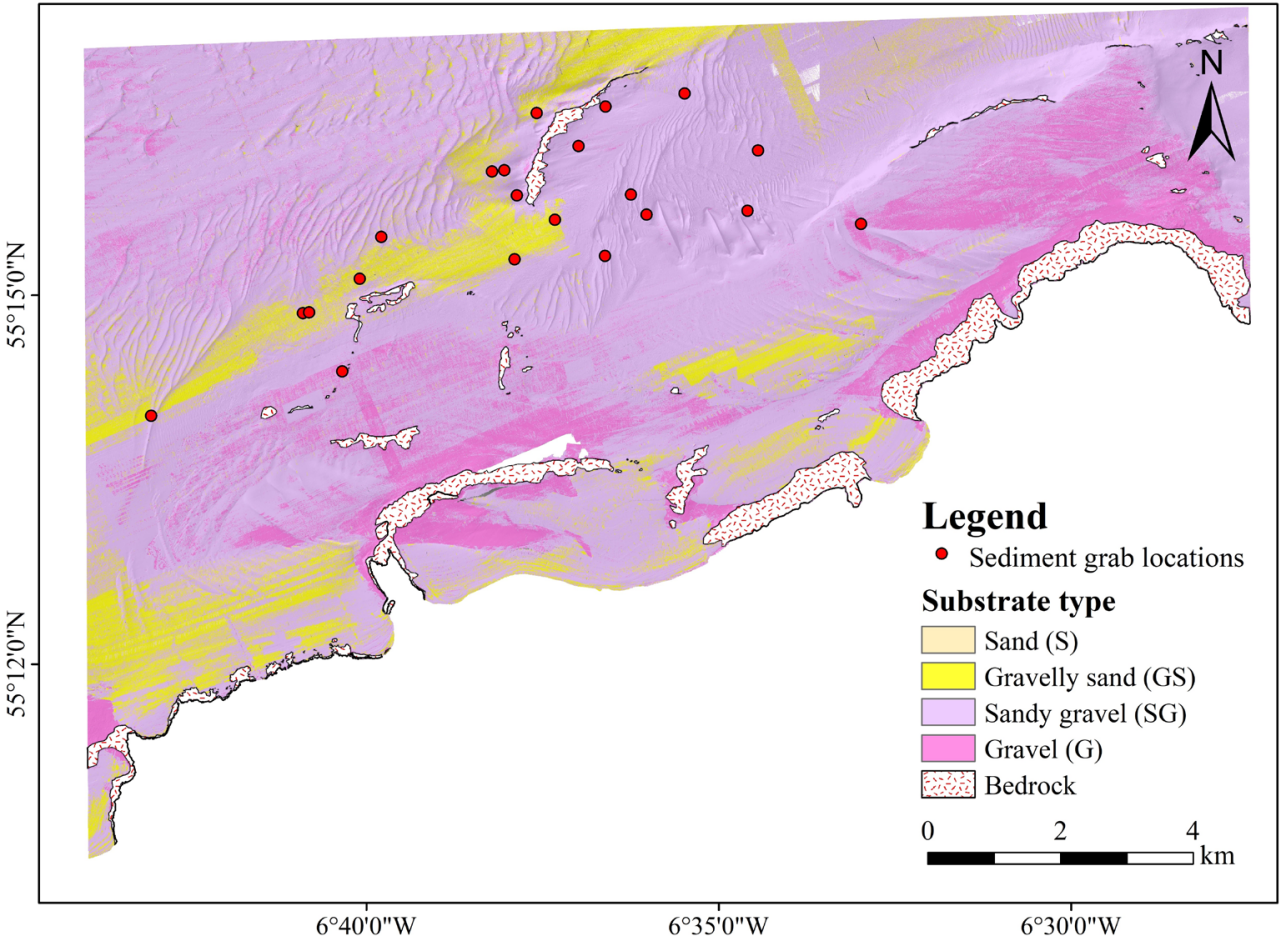
A substratum classification map of Skerries and Causeway SAC based on GLM (overlaid on a hillshade surface), and classified values of sediment grain size predicted based on Folk sediment classification; Sand (0.006–1.0 mm), Gravelly Sand (1.0–2.0 mm), Sandy Gravel (2.0–4.0 mm) and Gravel (4.0–9.0 mm).

A substratum classification map is generated for the SAC using MBES harmonised backscatter, bathymetry and bathymetry derivatives. The average bathymetric elevation (depth) within the SAC is −48.6 m (range: −1.7 m to −160 m) relative to Lowest Astronomical Tide (LAT), with a steep gradient away from the shore. It is determined that the relative backscatter intensity varies between −0.1 dB and −70to− dB (mean −28.7 dB) and that the slope angle varies between 0° and 86.7° (mean: 3.3°). Exploration analysis identifies slope angle and aspect as the variables that best characterise the terrain variability in the data. A manual classification aids the delineation of the seafloor area covered by bedrock (7.01 km^2^) from the dominant sediment region at 123.54 km^2^ (Figure 2).

The GLM model predicts sediment grain size ranging between 0.001 and 9 mm. The model evaluation yields an RMSE value of 970.4 and an adjusted *R* ^2^ of 0.44, as well as statistically significant results (*p < 0.05), and model coefficients summarised below (Table 1). When bathymetry and slope angle are independently fitted using backscatter, the RMSE values for the two models are 967.8 (adjusted *R* ^2^ = 0.4) and 965.3 (adjusted *R* ^2^ = 0.45). In addition, a moderate linear correlation was observed between backscatter and mean sediment grain size (*R* ^2^ = 0.46), while bathymetry (*R* ^2^ = 0.04) and slope (*R* ^2^ = 0.03) showed no meaningful correlation with sediment grain size. Overall, a mean grain size of 3.1 ± 0.8 mm is observed. Four substrate classes (sand, gravelly sand, sandy gravel and gravel) are realised from the predicted sediment grain size values using the Folk classification scheme as shown in Figure 2. Sandy gravel (SG) class comprising coarser sediments in the range 2–4 mm is the most dominant substrate, covering over 50% of the study area. The class represented by sand (S) with grain sizes between 0.001 and 1 mm has the lowest spatial coverage among the four predicted substrates. Along the shoreline is an extensive stretch of bedrock, which is a significant geomorphological feature of the area. It was found that the lengths and orientations of sand waves vary, indicating a considerable influence by a complex and dynamic hydrodynamic regime.

**TABLE 1:** Summary table of model performance and estimates implemented with generalised linear modelling with leave‐one‐out cross‐validation (LOOCV) method.

	Estimate	SE	*t*‐value	*p*
Intercept	9105.39	2624.89	3.47	0.003
Backscatter	180.94	43.59	4.15	0.001*
Bathymetry	15.86	16.68	0.95	0.354
Slope	−7.92	5.06	−1.57	0.134

* denotes statistical significance (*p* < 0.05).

### 3.2 Harbour Porpoise Tracking

**FIGURE 3:**
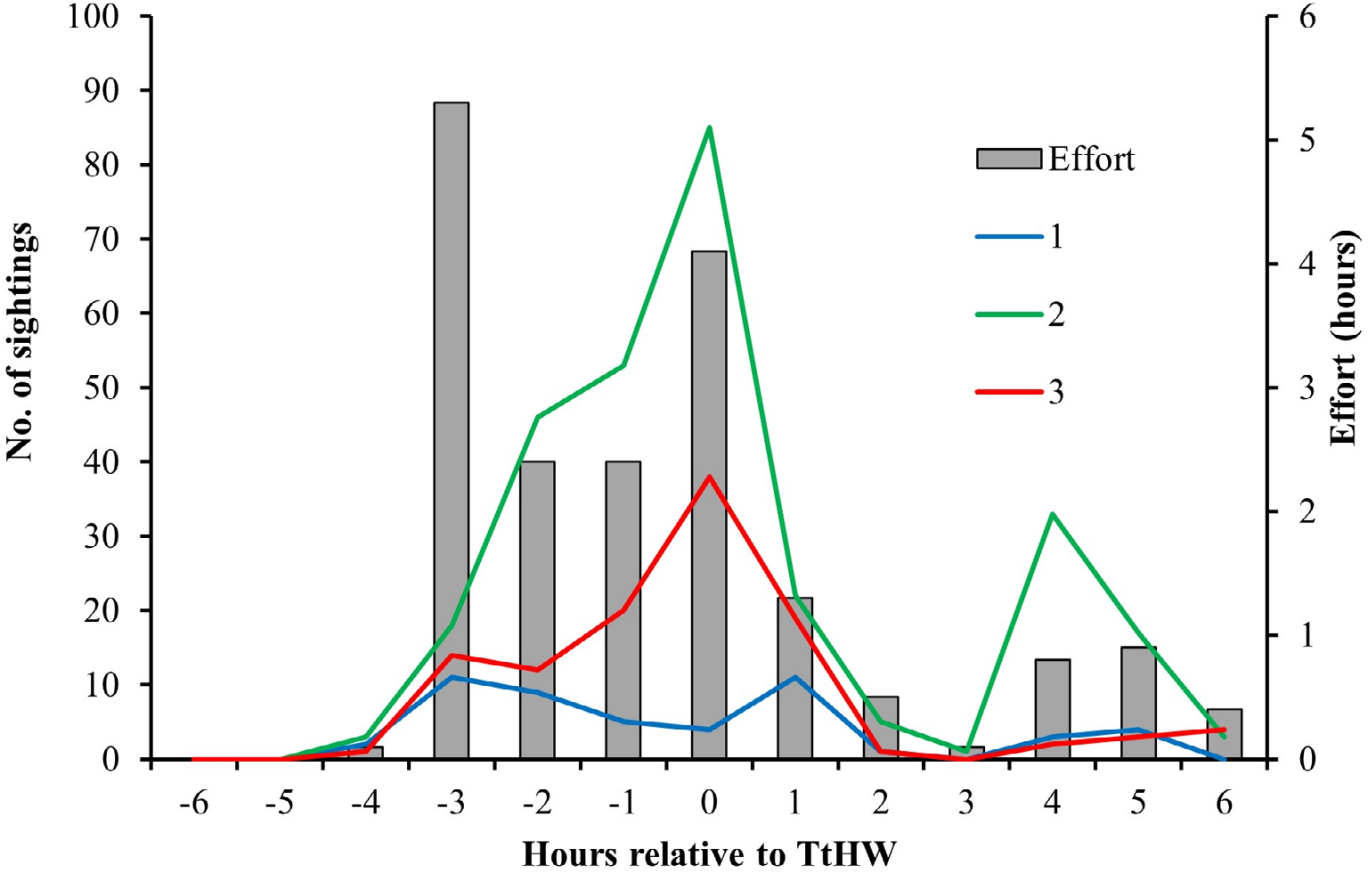
Number of porpoises' sightings from theodolite tracking for quality grades 1, 2 and 3 and survey effort (positive hours or active tracking) for hours relative to time to high water. The detection positive hours reflect the period during which a porpoise recording was made.

Between 6 and 17 August 2020, a total of 451 harbour porpoise sighting records were logged over the course of 11 days, with a total of 18.3 positive detection hours. Throughout the survey period, the number of porpoises' surfacing events detected varies significantly by hour relative to TtHW (Figure 3). The highest number of surfacing events is recorded between HW−3 and HW+3 h (*N* = 375) with a peak at high‐water time (*N* = 127). On the other hand, quality grade 2 has the highest number of sightings recorded (*N* = 286), followed closely by 3 (*N* = 114) and last 1 (*N* = 50). However, the effort (referring to active tracking, that is, positive detection hours when the animal was present) varies significantly across the hours relative to high water, with higher effort recorded before HW (10.1 h), followed by HW time (4.1 h) and hours after HW (4.1 h). The highest effort is concentrated at HW−3 (5.3 h) and HW (4.1 h), whereas HW−6, HW−5, HW−4, HW+1, HW+2 and HW+3 each have the lowest effort at < 1.5 h (Figure 3). The reduced effort at certain parts of the tidal cycle is primarily linked to weather‐related visibility constraints over the short study period and harbour porpoise movement patterns.

During the survey period, 42 porpoise sightings are recorded on average every day. The highest percentage of porpoise sightings occurs on 13 August 2020 (23%), followed by 7 August 2020 (15%). The final day of the survey period has the lowest porpoise sightings (0.43%). Part of this variability may reflect varying amounts of search effort across days rather than true changes in porpoise occurrence at the site. The tidal flow (tidal height), survey effort and the distance of harbour porpoise sightings to the shoreline have a substantial effect on their detection. In addition, exploratory research reveals that the hours relative to high water (TtHW) influence the relative porpoise sighting density, and all three are regarded to be additional environmental variables. However, a Pearson correlation between the relative density of porpoise sightings, and covariates reveals a weak association suggesting that the response variable is likely influenced in a non‐linear manner. The correlation between the density of porpoise sightings and tidal height (0.21, *p < 0.05), aspect (0.18, *p < 0.05) and nearest distance from the shoreline (0.25, *p < 0.05) was relatively stronger compared to the other covariates (< 0.1, *p < 0.05).

### 3.3 Density Clustering of Harbour Porpoise Sightings

**FIGURE 4:**
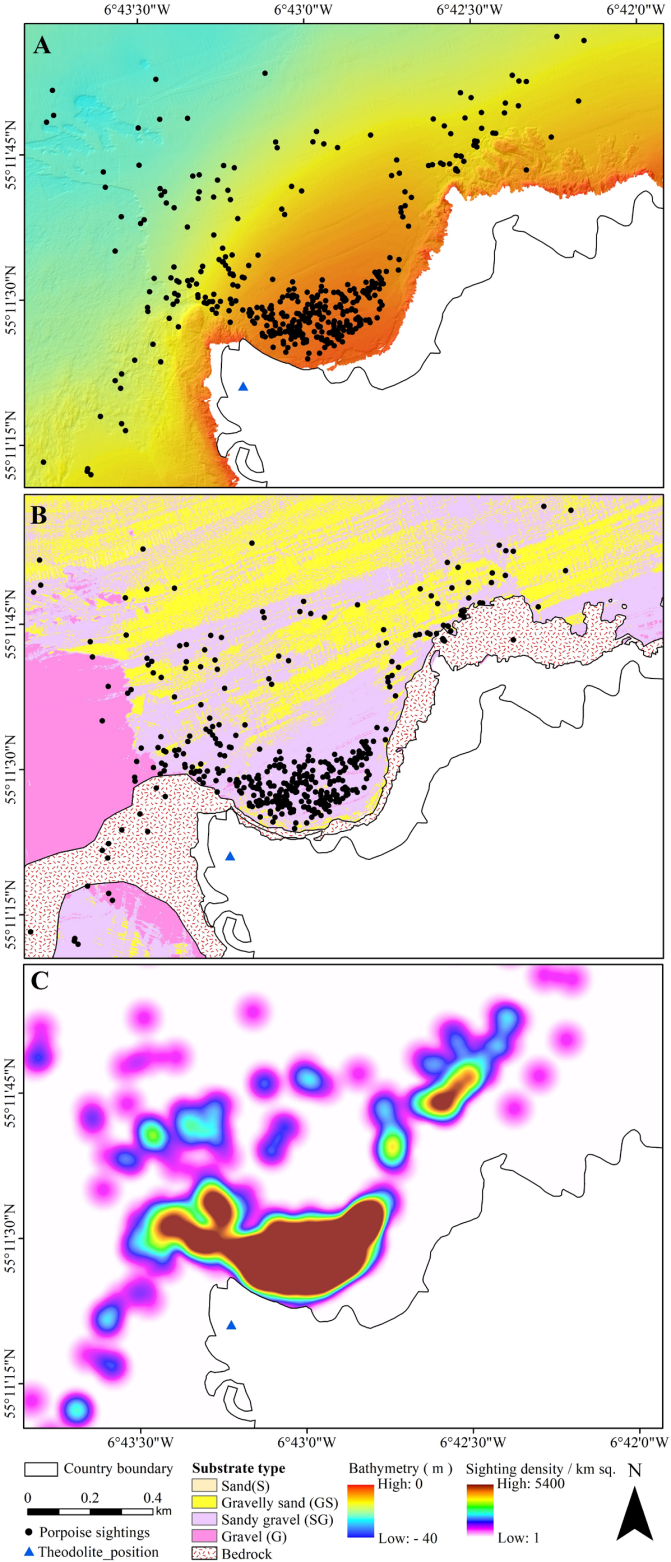
(A) Raw and filtered harbour porpoise sightings (*N* = 451) overlaid on the bathymetry grid at Portstewart point. (B) Harbour porpoise sightings overlaid on substrate map of the survey area showing the link between substrates and porpoise occurrence. (C) Displays a kernel density plot from the raw porpoise sightings by grid cell, produced using the ArcGIS' Spatial analyst extension (kernel density function) with a search radius of 1000 m and a spatial resolution of 1 m and displayed as the number of porpoise sightings per km^2^. The regions with dense clustering of harbour porpoise sightings are shown in brown colour.

Individual porpoise sightings (surfacing events) are converted to X and Y coordinates and overlaid on the bathymetry grid (Figure 4A) and substrate map (Figure 4B) around the survey site. These positions represent the raw porpoise sightings' records including multiple sightings/surfacing within a single observation of a porpoise recorded in different areas and days (even with the 10 min binning) and are filtered for detection approximation. However, delineating individual porpoises was not achievable. The results of the Kernel Density plot (Figure 4C) indicates that porpoises' sightings are concentrated closer to the shoreline. This result is linked to a detectability issue related to the shoreline position on visibility. We find that dense clusters in high occurrence areas also link sandy gravel (SG) and very shallow areas (low bathymetry). Dense clustering follows a similar pattern around headlands and the bedrock area of the seafloor, which dissipates with distance from the shoreline. The kernel density plot generated in ArcGIS at a spatial resolution of 1 m predicts an average density of 72 porpoise surfacing events per km^2^. Each porpoise position corresponding to a sighting is assigned an analysis window of 3 by 3 cells; this allows for the derivation of average values for each environmental variable and prevents spatial overlap caused by high‐level clustering, particularly near the high occurrence areas.

### 3.4 Modelling Harbour Porpoise Occurrence

**FIGURE 5:**
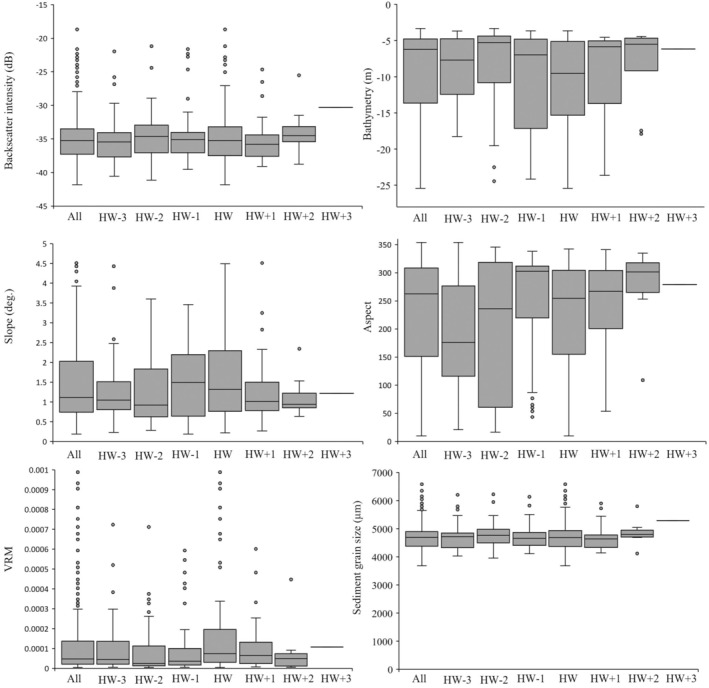
Environmental variability at the sampling location where porpoises were encountered across a half tidal cycle between 3 h before (HW−3) and 3 h after (HW +3) high water (HW). The combined environmental variability for all tidal phases is given as ‘All’.

**FIGURE 6:**
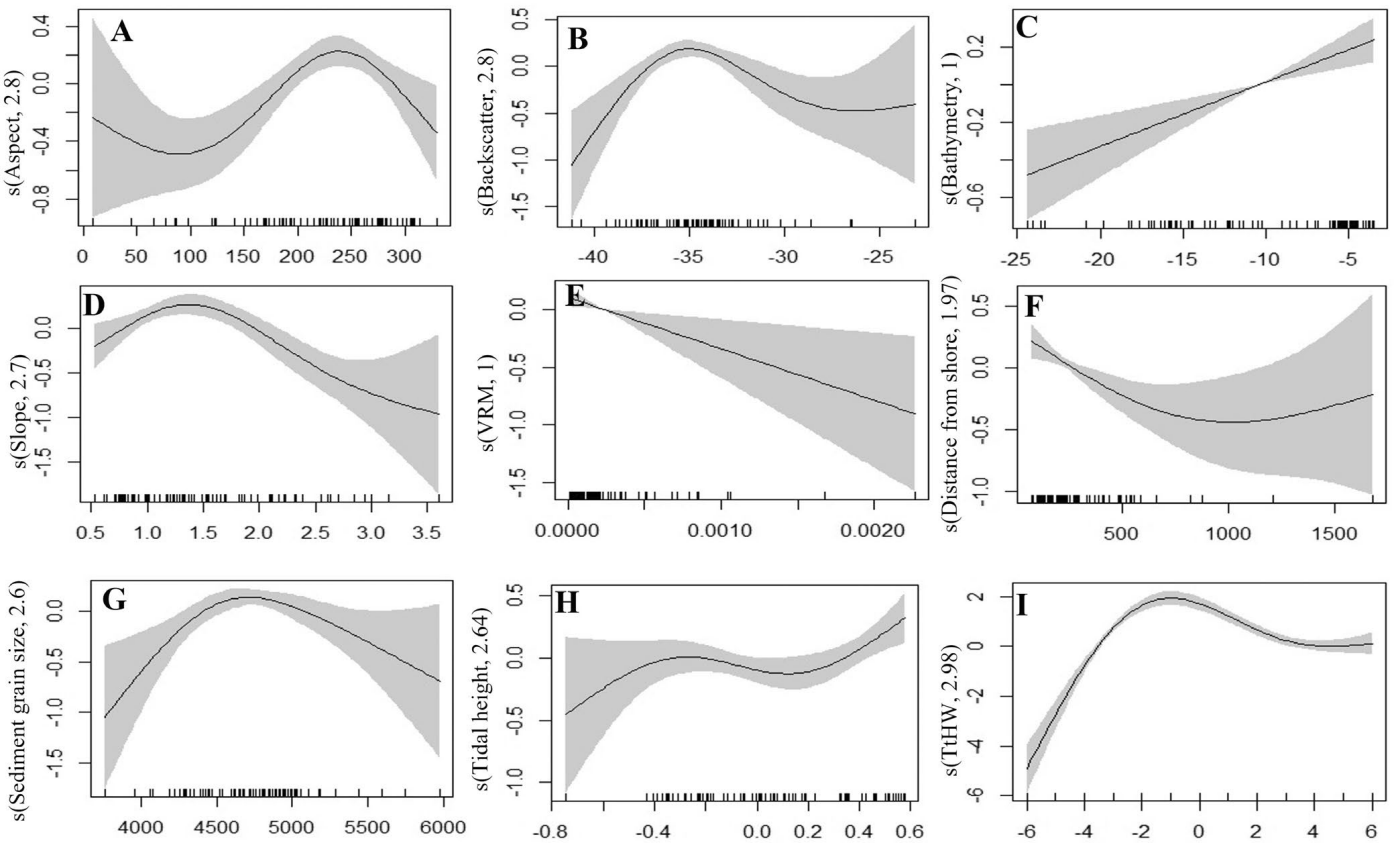
Harbour porpoise sightings distribution modelled as a function of hydrodynamic and environmental variables using a GAM with Poisson error structure with a thin plate regression spline (A). Smooth function of average aspect, (B) smooth function of average backscatter intensity, (C) smooth function of average bathymetry, (D) smooth function of average slope angle, (E) smooth function of terrain ruggedness measure_VRM, (F) smooth function of average nearest distance of porpoise sightings from the shoreline, (G) smooth function of average sediment grain size, (H) smooth function of average tidal height and (I) smooth function of hours relative to high water (TtHW). The shaded areas indicate the 95% confidence intervals and strips along the *x*‐axis represent the porpoise sighting data values.

Initial data analysis shows that acoustic signatures derived from MBES data relative to the positions of porpoises vary across all tidal phases (Figure 5). Slope, aspect and bathymetry vary widely, with the acoustic signatures for porpoises sighted before high water dominating. The porpoises' sightings are also found within a narrow range of backscatter, VRM and sediment grain size. In terms of the relative density of harbour porpoise sightings, the GAM models provide statistically significant outputs. The resulting final model explains 47.6% of the deviance, which is greater than the deviance explained by the individual univariate GAM models (Table 2). The most significant predictor variables for porpoise sightings were slope, backscatter intensity and aspect (*p < 0.05, Figure 6). Higher porpoise encounters are typically seen in regions with relatively steep slopes between 0.5° and 2° (Figure 6D) and shallow areas less than 17.5 m (Figure 6C). Backscatter intensity in the range –40 dB and −30 dB sees a higher porpoise occurrence (Figure 6B). On the other hand, higher aspect values are observed to support a higher density of porpoise sightings.

**TABLE 2:** Summary of *N* = 451 recorded harbour porpoise sightings analysed using GAM with a Poisson error structure and thin plate regression splines.

RI	Linear term/smooth (df)	%Deviance	Adjusted *R* ^2^	AIC	χ^2^	Estimated *p*_value
1	S(Slope), edf = 2.7	15.8	0.10	447.5	24.5	< 0.05*
2	S(Backscatter), edf = 2.8	11.8	0.07	455.5	18.1	< 0.05*
3	S(Aspect), edf = 2.8	11.3	0.07	456.6	19.8	< 0.05*
4	S(Grain size), edf = 2.6	9.3	0.05	460.3	16.0	< 0.05*
5	S(Bathymetry), edf = 1	8.7	0.07	457.8	16.1	< 0.05*
6	S(TtHW), edf = 3.0	8.1	0.05	461.0	12.9	< 0.05*
7	S(Distance to shore), edf = 2.0	6.9	0.04	464.0	11.4	< 0.05*
8	S(Tidal height), edf = 2.6	6.7	0.04	465.5	12.3	< 0.05*
9	S(VRM), edf = 1	4.4	0.02	466.3	7.3	< 0.05*
Final	S(TtHW) + S(Grain size) + S(Backscatter) + S(Aspect) + S(Slope) + s(Bathymetry) + S(Tidal height + S(Distance to shore)	47.6	0.41	408.9		

*Note:* The selected variables are listed in order of relative importance (RI) for the eight covariates based on increased deviance explained by each covariate and a relative decrease in the AIC score used to select the final model. At *p* < 0.05, all univariate models including the final model produced statistically significant output. The ‘edf’ represents the degrees of freedom of the estimated smooth functions.

* denotes statistical significance (*p* < 0.05).

The relative density of porpoise sightings is greatest between HW−3 and HW+3 h (Figure 6I), with lower sighting densities recorded 5 h before and after TtHW, and the lowest density occurring 6 h before TtHW. While this variability in sightings is partially explained by variation in survey effort per hour relative to high water, GAM modelling indicates that tidal height and hydrodynamic conditions still play a role, though their influence is relatively weak compared to substrate and topographic features. The highest number of individual theodolite measures (N) was taken around HW+3 and HW−3 (*N* = 375) because this is when the most encounters (positive hours) occurred. Outside of this tidal window, a low effort (2.2 h) is devoted to porpoises' observation, resulting in the fewest sightings recorded (*N* = 76). Specifically, the HW−3 and HW tidal phases have the greatest effort at 5.3 and 4.1 h, respectively, but the HW phase had the most sightings at 127 (Figure 3).

The GAM model demonstrates a statistically significant relationship between predicted sediment grain size and porpoise sighting density, with higher porpoise occurrences favoured in locations with coarser sediments (4.25–5 mm), which correspond to sandy gravelly sediments. In contrast, decreasing densities in porpoise sightings are observed in finer sediments below 4 mm and gravelly sediments above 5 mm (Figure 6G), indicating a specific substrate preference influencing porpoise distribution. The coarse substrate types (sandy gravel) and bedrock dominate the area with the highest occurrence and dense clusters of porpoise sightings, which correspond to hydrodynamic features, such as headlands, eddies and slope gradient (Figure 4). Sediment grain size reflects the energy at that point on the seafloor where coarser sediments (higher backscatter intensities) and finer sediments (lower backscatter intensities) are found in higher and lower flow regimes, respectively. With deviance of 6.7% and 4.4%, respectively, the tidal height (Figure 6H) and VRM (Figure 6E) are the least important predictor variables with respect to porpoise sighting density. A higher concentration of sightings occurs between −0.4 m and 0.2 m and shows an increasing trend towards 0.6 m of tidal height (Figure 6H). Generally, low VRM values are linked with a higher clustering of porpoises. The highest density of porpoise sightings is reported near the shoreline (within ~500 m), with occurrence decreasing further away from the shore (Figure 6F).

**FIGURE 7:**
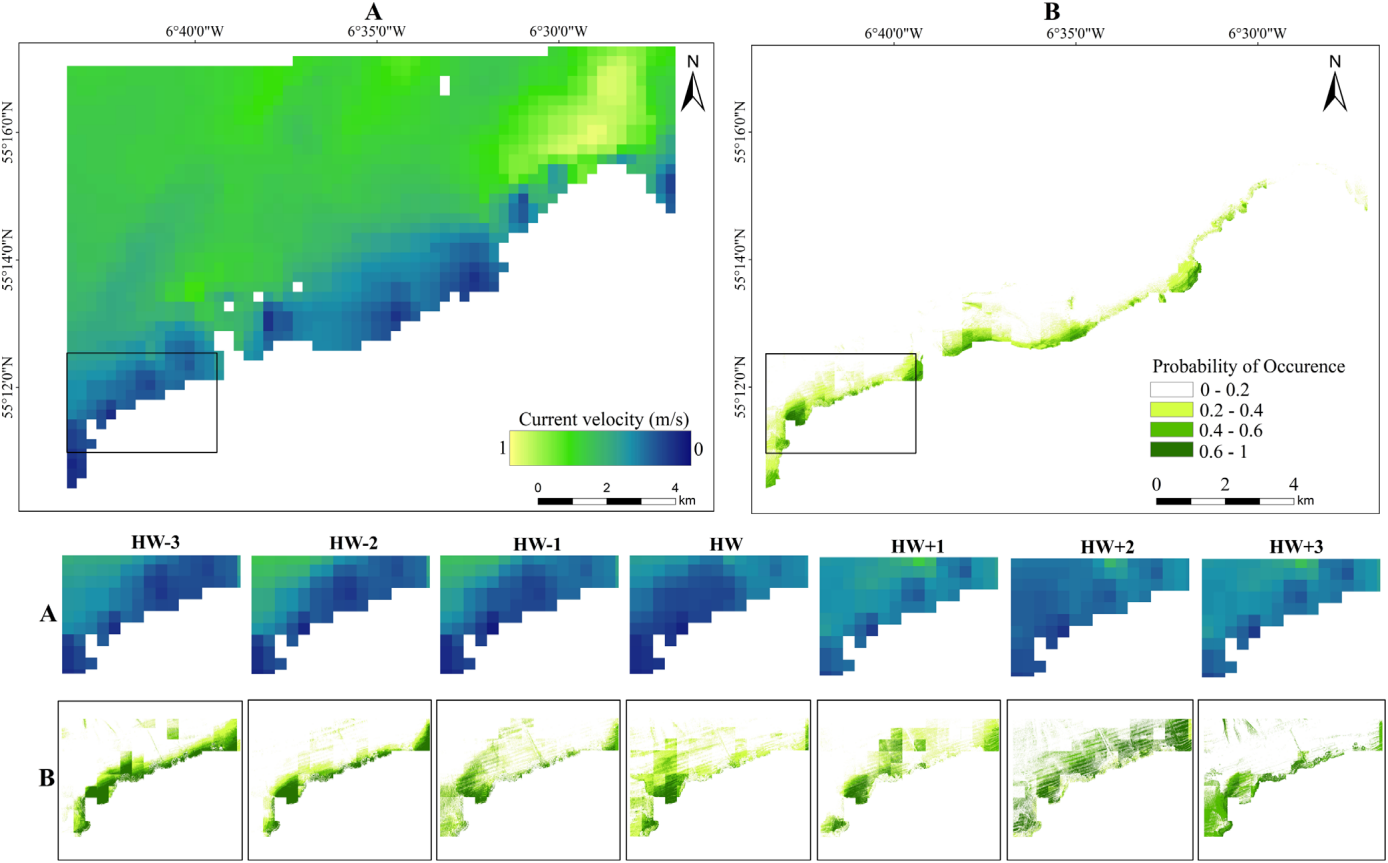
(A) Average current speeds (1–0 m/s) and (B) predicted probability of occurrence surfaces along a half tidal cycle (+3 to −3 h) relative to high water during the survey period of August 2020 for the full coverage (top) and an inset (bottom). The labels on the top of the inset panels (HW−3–HW + 3) represent the hours relative to high‐water time for both neap and spring tidal phases. For probability occurrence surfaces, greener areas indicate areas with a higher likelihood of encountering harbour porpoises (> 0.6), whereas whitish areas are those where porpoises are less likely to be detected (< 0.2).

The probability of occurrence’ models show a minimal variation across different hours relative to TtHW, but highlights higher probability of porpoise occurrence (> 0.6) in coastline areas linked to headlands with bedrocks and coarser sediments. However, comparing these high occurrence areas for models before (HW−3, HW−2, HW−1) and after high water (HW +3, HW +2, HW +1) reveals a significant variation in their spatial extent (Figure 7, Figure A1, Figure B1). Generally, the results demonstrate that a relatively higher current speed away from the shoreline is associated with lower probability of occurrence of porpoises (Figure 6). Overall, while survey effort influences sighting density patterns, GAM modelling suggest that substrate type, hydrodynamic conditions and local topography are stronger predictors of porpoise occurrence than tidal height alone. Model evaluation reveals that including hydrodynamic inputs (current velocity) improves the overall model fitting from an average of 0.96 AUC value (deviance = 0.19, TSS = 0.75) to 0.98 (deviance = 0.14, TSS = 0.83). HW−2 is the best fitted model yielding AUC, TSS and deviance values of 0.99, 0.89 and 0.15, respectively. Meanwhile, HW+2 is the poorly fitted model compared to the other models with AUC value of 0.82 (deviance = 2.62, TSS = 0.59).

## 4 Discussion

This study establishes a significant relationship between local hydrodynamic and environmental conditions, and the relative density and probability of occurrence of porpoises in a local flow acceleration around a headland. The findings demonstrated the utility of a multidisciplinary analysis approach in examining the linkages between environmental conditions and a species of conservation interest. In particular, the GAM models yielded statistically significant findings identifying slope, aspect and backscatter intensity as the most important environmental variables in determining relative porpoise sighting density. In general, the analysis revealed that the use of space by porpoises in closely spaced theodolite tracking recordings was highly variable. Again, the probability of occurrence indicates significant spatial variation where near‐shore areas, specifically headlands comprised of bedrock and coarser sediments found on the seafloor, are linked to high frequent encounters of porpoises. The results are key to understanding the underlying processes that influence harbour porpoises' habitat use of space and occurrence.

### 4.1 Substratum Classification

Skerries and Causeway is an SAC designated due to the presence of Annex I habitats reefs, sandbanks and sea caves, and Annex II species harbour porpoise *Phocoena phocoena* (Goodwin et al. 2012; Plets et al. 2012). This project initially created a substratum map using statistically compensated MBES backscatter (harmonised backscatter) and a modelling approach. Harmonised backscatter, which provides a statistical calibration for multiple and overlapping data sets from different MBES systems and surveys, has been used more recently (Misiuk et al. 2021, 2020). While harmonising backscatter data, two fundamental assumptions are made: (i) the backscatter values of each data set are a result of the same substrate type and (ii) there should be a sufficient degree of temporal homogeneity between data sets (Misiuk et al. 2020).

The Skerries and Causeway SAC is dominated by coarse sediments (> 2 mm), with sandy gravel (2–4 mm) covering slightly over 50% of the study area (Figure 2). These coarse sediments are characterised by strong hydrodynamic conditions and higher backscatter intensity (Evans et al. 2015; Plets et al. 2012), which may influence foraging behaviour and prey availability for harbour porpoises (Benjamins et al. 2017). Coarse sediments are often linked with high‐energy environments that are subsequently associated with enhanced prey aggregation, particularly small fish species that form an important component of the harbour porpoise diet (Greene et al. 2020; Benjamins et al. 2017). The presence of bedrock and coarse substrates near headlands and tidal environments may help engineer the formation of localised upwelling zones and eddies, further influencing prey distribution (Waggitt et al. 2018; Lewis et al. 2015). Understanding specific habitat characteristics is important for not only assessing critical foraging areas but also for designing and implementing targeted conservation strategies for harbour porpoises within and beyond SAC boundaries.

### 4.2 Harbour Porpoise Tracking

During the 11‐day survey period, a total of 451 porpoise sightings were recorded, with 18.3 detection positive hours attained. During the survey, it was noted that the variable survey effort related to visibility conditions, tidal phase, and distance to shore had a significant effect on harbour porpoises' positive detection.

While seasonality was not accounted for in this study due to resource limitations, previous research on seasonal trends has demonstrated that the site occurrence of harbour porpoises is particularly highest in the summer months (Waggitt et al. 2019). Understanding seasonal patterns is crucial for predicting temporal shifts in porpoise distributions and informing the development of adaptable conservation measures. Harbour porpoises are also known to spend 75% of their time hunting—alone or in groups—utilising tidal‐energy regions that favour prey aggregation and, as a result, boost foraging opportunities (Schaffeld et al. 2016). To evaluate the underlying biophysical processes that are primarily responsible for the spatial and temporal distribution of porpoises, it is crucial to examine the use of space and environmental conditions (Benjamins et al. 2017, 2016; Jones et al. 2014; Booth et al. 2013). Low Pearson correlation coefficient values were found between relative porpoise density and environmental variables. The correlation between tidal height, aspect and nearest distance to the shoreline was relatively higher than other covariates, between 0.15 and 0.25.

### 4.3 Relationship Between Harbour Porpoises and Abiotic Proxies

It has been observed elsewhere that harbour porpoises utilise periods of increased tidal activity (Nuuttila et al. 2017; Lewis et al. 2015). In the present study, this is likely due to the interaction between tidal currents and the sharp bathymetric gradient around Portstewart point. Enhanced tidal activity enhances prey material (e.g., zooplankton, small forage fish) circulation, hence generating ideal foraging conditions, particularly along headlands (Jones et al. 2014). However, the relationship between harbour porpoise as a predator and prey aggregations is complex, poorly understood and believed to be associated with optimal foraging efficiency (Ransijn et al. 2021; Schaffeld et al. 2016; Heinänen and Skov 2015; Sims et al. 2008). Even while it was out of the scope of this study to examine prey–predator relationships, it cannot be ruled out that the availability and distribution of prey species such as sandeels, cod, whiting, Atlantic herring, squids and shrimps, which form part of the harbour porpoise diet, could have an influence on their spatial distribution patterns (Stedt et al. 2024; Ransijn et al. 2021; Sveegaard et al. 2012).

The GAM model revealed that predicted sediment grain size explained 9.3% of the deviance (*p < 0.05) and indicated that coarser sediments in the range 4.25–5 mm (sandy gravel class) supported a higher density of porpoise sightings than finer sediments. Sandeels, which is one of the prey species for porpoises have an affinity for coarse sediments than other prey species, which may have ramifications for how these data are interpreted (Greene et al. 2020; Tien et al. 2017). In addition, porpoise sightings were higher in regions with relatively higher backscatter intensity. Backscatter intensity is a surrogate for material property and seafloor roughness, which is partially dependent on the area's substrate type and frequency response (Runya et al. 2021; Gaida et al. 2018). Results indicate that increased seabed roughness influences marine animal presence, particularly at shallow depths (Lieber et al. 2018). Habitat variability resulting from these substrates is typically correlated with higher prey populations, making these regions optimal for porpoise’ foraging (Benjamins et al. 2017). Future research of this nature would benefit from the incorporation of multi‐frequency backscatter that can capture the frequency response of both the physical environment and the biological communities that comprise the volume scattering (Runya et al. 2024; Brown et al. 2019; Gaida et al. 2018; Montereale Gavazzi et al. 2016).

According to the GAM models, the VRM, tidal height and distance to the nearest shoreline were the least significant variables. However, porpoises were observed to be more concentrated near the shore, within ~500 m from the shoreline. Potentially, the distance from the shoreline or land could serve as a proxy for other vital environmental variables associated with porpoise distribution such as water circulation and frontal systems, and their subsequent influence on the availability of prey opportunities (Rojano‐Doñate et al. 2024; Booth et al. 2013). The presence of headlands close to the shoreline facilitates the formation of upwelling features, fronts and eddies, which may result in a higher prey density close to these features and provide porpoises with greater foraging opportunities (Greene et al. 2020; Benjamins et al. 2015). These features are also present further north in Portrush and Giant Causeway, which are potentially ideal to support occurrence and activity of porpoises. Again, visibility of porpoises is likely to be enhanced near the shoreline than far away pointing to a detectability issue in the results.

In addition, shallow bathymetry (< 20 m) and rather low slope angle (< 2°) were associated with an increased porpoise sighting density. These findings are consistent with past studies that established comparable patterns mostly associated with moderate depths (Jones et al. 2014; Marubini et al. 2009), slope and distance to shore (Booth et al. 2013), and tidal regime (Nuuttila et al. 2017). It is recognised that the slope of the seafloor influences porpoise distribution, with more sightings occurring in places with a steepening slope (Booth et al. 2013; Embling et al. 2010). Together, slope and shear stress influence the circulation of currents and thus the biological productivity that attracts prey species (Inall et al. 2009).

### 4.4 Probability of Occurrence

Previous studies have indicated that hydrodynamic conditions significantly influence site occurrence of harbour porpoises, particularly in and around near‐shore locations associated with coarse sediments and strong tidal currents (Benjamins et al. 2017, 2015). The models showing the probability of occurrence in this study align with the findings of previous research, suggesting higher porpoise presence (> 0.6 probability) in coastal areas, particularly around headlands, where hydrodynamic conditions may enhance prey availability. Hydrodynamic forcing across shallow topography and vertical displacement around headlands can result in localised prey aggregation, enhancing foraging resources' availability for harbour porpoises (Lieber et al. 2018). Similar patterns have been observed previously, where enhanced flow regimes have the ability to generate foraging opportunities for small cetaceans (Benjamins et al. 2015; Heinänen and Skov 2015; Embling et al. 2013). Besides, model validation underpins the influence of hydrodynamics in predicting harbour porpoise occurrence, as the inclusion of current velocity improved model performance from an AUC of 0.96 (TSS = 0.75) to 0.98 (TSS = 0.83). Again, this is consistent with past research highlighting the ecological role of hydrodynamically‐influenced regions as favourable foraging grounds for harbour porpoises (Benjamins et al. 2017).

The Skerries and Causeway SAC designation under the EC Habitats Directive (Council Directive 92/43/EEC) primarily considers Annex 1 habitats (reefs, sandbanks, sea caves) and Annex II species ( *P. phocoena* ) as legally protected features. The conservation objectives under this framework aim to maintain the ecological integrity of the site for harbour porpoises, while protecting them from significant disturbance and preserving supporting habitats and prey resources (DAERA 2025; JNCC 2022). This study demonstrates that harbour porpoises actively forage within and around headlands, a key ecologically significant area that extends beyond the current SAC boundary. From a management perspective, the high mobility of this species enables it to move both within and outside the designated SAC, highlighting a need for effective management strategies that take into account the dynamic nature of this species' use of space beyond static legal boundaries. These findings reinforce the idea of considering fine‐scale environmental variability when assessing harbour porpoises' use of space and underline the importance of evaluating conservation strategies at a more localised scale relative to ecosystem‐wide management approaches and existing legal frameworks. While a huge burden is placed on law enforcement to achieve these conservation objectives, and regular monitoring and reporting (i.e., every 6 years), integrating spatially adaptive management approaches could further enhance the protection of harbour porpoises and their associated prey species.

### 4.5 Limitations and Future Work

In these types of modelling studies, the lack of temporally appropriate ground truth data for validation remains a key impediment, with most research depending on extant data. Despite this challenge, the analysis workflow and findings in this study are important for providing a basis for informing the design of future surveys and subsequent workflows for multidisciplinary analysis for marine mammal research. Visual surveys based on a single theodolite station reveal site‐specific patterns inside the observer's line of sight and eventually limit the geographical scope of the sampling effort to nearshore areas. Visual surveys can under‐ or over‐sample porpoises due to their size, less conspicuous surface behaviour and disproportionate survey effort (Nuuttila et al. 2017). To address detectability issues and improve model robustness, future sampling designs should be able to account for species‐specific life history strategies, seasonality and tidal dynamics. Future research on harbour porpoises at this site should ensure systematically distributed effort across all tidal phases to minimise effort‐related bias. Besides, targeted dietary studies, including assessments of sandeel presence and other potential prey species (e.g., whiting, cod, shrimps), would provide a better understanding of prey–predator dynamics and evaluate the relative significance of different forage resources. In general, the findings of this study represent a robust workflow for establishing a species‐specific monitoring strategy and provide insights that can support marine spatial planning around features (e.g., headlands) that support higher concentrations and subsequent effective conservation of harbour porpoises.

## 5 Conclusion

This study demonstrates the utility of a multidisciplinary analysis integrating local hydrodynamic and environmental variables in assessing harbour porpoises' ( *Phocoena phocoena* ) site occurrence and use of space. Generalised Additive Modelling (GAM) identified slope, aspect, backscatter intensity and sediment grain size as key predictors of porpoise sighting density. The findings particularly indicate the role of areas linked to shallow bathymetry, low slope, coarser sediments and proximity to the shoreline in supporting higher porpoise occurrence and space use, especially in areas of local flow acceleration around headlands. This study highlights the short‐term variability in time and space across hours and tens of meters in porpoise use of space, demonstrating dynamic habitat selection. While this study is limited to a short 11‐day survey period, it provides a robust workflow for developing a species‐specific monitoring strategy for harbour porpoises. Monitoring this European Protected Species (EPS) in the future may need the incorporation of seasonality, passive acoustic monitoring, prey dynamics and adaptive sampling techniques to improve model accuracy and detectability. The findings further highlight the importance of fine‐scale habitat features, particularly headland‐associated flow accelerations, which may be linked to porpoise occurrence both in and out of Special Areas of Conservation (SAC). By leveraging multidisciplinary methodological approaches, this study provides a scientific basis for refining marine conservation strategies, delivering long‐term protection of harbour porpoises' habitats under existing legal and management frameworks.

## Author Contributions

**Robert Mzungu Runya:** conceptualization (lead), data curation (lead), formal analysis (lead), funding acquisition (equal), investigation (lead), methodology (lead), project administration (lead), software (lead), supervision (lead), validation (lead), visualization (lead), writing – original draft (lead), writing – review and editing (equal). **Chris McGonigle:** conceptualization (equal), formal analysis (supporting), funding acquisition (equal), investigation (equal), methodology (equal), project administration (supporting), supervision (lead), validation (supporting), writing – original draft (supporting), writing – review and editing (equal). **Rory Quinn:** conceptualization (supporting), formal analysis (supporting), investigation (equal), methodology (equal), project administration (supporting), supervision (lead), validation (supporting), writing – original draft (supporting), writing – review and editing (equal). **Morgane Pommier:** formal analysis (supporting), funding acquisition (supporting), investigation (equal), writing – original draft (supporting), writing – review and editing (equal). **Christian Armstrong:** data curation (equal), formal analysis (supporting), investigation (equal), methodology (supporting), writing – original draft (supporting), writing – review and editing (equal).

## Ethics Statement

Theodolite tracking is a totally passive and non‐contact monitoring method. Animals were observed from shore through a telescope, never approached and not tagged with physical devices (a contrast to satellite/GPS tracking).

## Conflicts of Interest

The authors declare no conflicts of interest.

## Supporting information


Data S1.

## Data Availability

The multibeam data and particle size analysis sediment data used is publicly available on https://experience.arcgis.com/experience/3f2815ec89e745d2b65630429d06385c and https://data.marine.ie/geonetwork/srv/eng/catalog.search#/metadata/ie.marine.data:dataset.5029, respectively. Meanwhile, the tidal data are available upon request at https://www.bodc.ac.uk/data/hosted_data_systems/sea_level/uk_tide_gauge_network/#time_series. Additionally, Data S1 has been provided comprising the field tracking observations of harbour porpoise's species *Phocoena phocoena* .
